# Serological diagnosis of pneumocystosis: production of a synthetic recombinant antigen for immunodetection of *Pneumocystis jirovecii*

**DOI:** 10.1038/srep36287

**Published:** 2016-11-08

**Authors:** A. L. Tomás, F. Cardoso, F. Esteves, O. Matos

**Affiliations:** 1 Unidade de Parasitologia Médica, Grupo de Protozoários Oportunistas/VIH e Outros Protozoários, Instituto de Higiene e Medicina Tropical, Global Health and Tropical Medicine, Universidade Nova de Lisboa, Lisboa, Portugal; 2 Centro de Toxicogenómica e Saúde Humana (ToxOmics), Departamento de Genética, NOVA Medical School/Faculdade de Ciências Médicas, Universidade Nova de Lisboa, Lisboa, Portugal

## Abstract

Diagnosis of *Pneumocystis* pneumonia (PcP) relies on the detection of *P. jirovecii* in respiratory specimens obtained by invasive techniques. Thus, the development of a serological test is urgently needed as it will allow the diagnosis of PcP using blood, an inexpensive and non-invasive specimen. This study aims to combine the production of a multi-epitope synthetic recombinant antigen (RSA) and an ELISA test for detection of anti-*P. jirovecii* antibodies, in order to develop a new approach for PcP diagnosis. The RSA was selected and designed based on the study of the immunogenicity of the carboxyl-terminal domain of the major surface glycoprotein. This antigen was purified and used as an antigenic tool in an ELISA technique for detection of Ig, IgG and IgM antibodies anti-*P. jirovecii* (patent-pending no. PT109078). Serum specimens from 88 patients previously categorized in distinct clinical subgroups and 17 blood donors, were analysed. The IgM anti-*P. jirovecii* levels were statistically increased in patients with PcP (*p* = 0.001) and the ELISA IgM anti-*P. jirovecii* test presented a sensitivity of 100% and a specificity of 80.8%, when associated with the clinical diagnosis criteria. This innovative approach, provides good insights about what can be done in the future serum testing for PcP diagnosis.

*Pneumocystis jirovecii* is an atypical opportunistic fungus capable of causing severe interstitial pneumonia that remains the leading AIDS-defining illness in European countries and the USA^1^,^2^,^3^. Furthermore, PcP is an emerging concern in immunosuppressed non-HIV-infected patients subjected to immunosuppressive therapies due to cancer, organ transplant or autoimmune diseases^4^,^5^.

The standard laboratory diagnosis of PcP relies on microscopic visualization of stained *P. jirovecii* organisms and/or DNA detection by PCR in respiratory specimens, such as bronchoalveolar lavage (BAL). These specimens are obtained by invasive techniques that carry an associated risk of complications and are not easy to perform in patients with respiratory failure or in children^6^. Therefore, development of a serological test is urgently needed, as it will allow the diagnosis of PcP using minimally invasive samples, such as blood.

In the past few decades, many serological methods have been studied for use in the diagnosis of PcP. Elevated serum levels of the lactate dehydrogenase (LDH), (1–3)-β -D-glucan and Krebs von den Lungen-6 antigen (KL-6), as well as low serological levels of S-adenosylmethionine (SAM), have been related to PcP and proposed as markers of the disease^7^,^8^,^9^,^10^,^11^,^12^,^13^,^14^,^15^. However, the use of these metabolites in PcP diagnosis is complex because their serum levels are not strictly specific to *P. jirovecii* infection.

Alternatively, promising studies using recombinant antigens of *P. jirovecii* and antibody immunodetection techniques, such as immunoenzymatic or immunoblotting assays, have shown potential application in the diagnosis and epidemiological studies of PcP^16^,^17^,^18^,^19^,^20^,^21^,^22^,^23^,^24^,^25^,^26^. The *Pneumocystis* antigen that has received the most attention is the major surface glycoprotein (Msg), which contains shared and species-specific epitopes, elicits humoral and cellular protective immune responses and plays a central role in the interaction of *Pneumocystis* with its host^27^. The carboxyl-terminal domain of the Msg is reported to be highly immunogenic, the most conserved and reactive region of Msg and to contain both B and T cell conserved protective epitopes^16^,^18^,^23^,^24^,^25^. Therefore, recombinant synthetic amino acid sequences, designed to hold more than one reactive region of the Msg, are promising tools that can increase the sensitivity, specificity, the cost-effectiveness and the standardization of serological tests^28^.

This study aims to: (1) produce a multi-epitope synthetic recombinant antigen (RSA) of *P. jirovecii*’s Msg; (2) optimize an indirect enzyme-linked immunosorbent assay (ELISA) method for the detection of anti-*P. jirovecii* Ig, IgM and IgG antibodies, in order to use the *P. jirovecii* RSA as biosensor in the serologic diagnosis of PcP.

## Materials and Methods

### Clinical samples

A total of 105 serum specimens were analysed in a retrospective observational study with the purpose to evaluate the reliability of the ELISA developed. Eighty-eight sera were from patients attending hospitals in the Lisbon area, between 2010 and 2013, whose specimens were submitted to our laboratory with the purpose of routine diagnosis of PcP with the patients’ informed consent and according to the routine institutional procedures. Patients’ demographic data were kept in confidence and were coded for the authors of the study. Only laboratory results, clinical diagnosis of PcP and immunosuppression status were revealed and are summarized in Table 1. Seventeen blood donors’ serum specimens were studied to provide a control group.

The clinical diagnosis of PcP was establish when at least two of the following variables were present: symptoms such as unproductive cough, fever and dyspnea; arterial partial pressure of oxygen (PaO_2_) lower than 65 mmHg; chest radiographs presenting fine bilateral, perihilar interstitial shadowing^6^,^29^,^30^.

*P. jirovecii* burden was quantified by scoring the number of cysts observed in respiratory specimens by applying the semi-quantitative method of IF/Mab and was defined as: low to moderate (one to three cysts in one field at x1,000), and heavy (four or more cysts in one field at x1,000), as described previously^31^.

The serum specimens enrolled in this study were from patients’ previously categorized in five distinct clinical subgroups by detection of *P. jirovecii* in their respiratory specimens by indirect immunofluorescence with monoclonal antibodies (IF/MAb) and nested-PCR (nPCR), as described previously^32^. Serum specimens from patients with PcP (positive IF/MAb, positive nPCR), patients colonized with *P. jirovecii* (negative IF/MAb, positive nPCR, asymptomatic), patients without *P. jirovecii* or other fungal infections (negative IF/MAb and nPCR), patients without *P. jirovecii* infection but with other fungal diseases (negative IF/MAb and nPCR, presence of other fungi) and from blood donors (healthy persons), were analysed.

The protocol for this study was approved by the ethical committee from Instituto de Higiene e Medicina Tropical (Lisboa, Portugal) that, because this was a retrospective observational study, waived informed consent. The methods were carried out in accordance with the approved guidelines.

### Selection and cloning of the recombinant synthetic antigen (RSA) sequence

The multi-epitope RSA was designed based on the study of the immunogenicity of the *P. jirovecii’s MSG* gene, described previously (*GenBank* accession no. AF033211 and JN792933.1)^33^,^34^. The selection of the putative reactive epitopes of the Msg was made targeting the terminal fraction of its middle portion (MsgB) and all its terminal portion (MsgC), using bioinformatics approaches to analyse electrochemical properties, secondary structure prediction, polarity, relative position to the membrane and hydrophobicity profile of the specific polypeptides at the online software *ExPASy – ProtScale* and *CBS – TMHMM* – version 2.0, as previously described^28^. In addition, only the most conserved regions, presenting high similarity with the sequences previously reported in GenBank for *Pneumocystis*’ Msg protein, were considered for the final selection of the RSA.

Three potential immunogenic regions with high predicted antigenicity and reactivity were chosen and a short oligonucleotide sequence containing these three epitopes, interconnected by two bridges of five glycine residues, was synthesized and cloned into the plasmid pUC57-Amp vector by Nzytech^®^. The inserts sequence was confirmed by sequencing (ABI 3730XL sequencer, Stabvida^®^). Several physical and chemical parameters of the RSA were determined using the online tool ExPASy – ProtParam.

After the RSA synthesis process into pUC57-Amp, the vector was cloned into CaCl_2_ competent *E. coli* TOP10 by heat shock, in a proportion of 0.1 μg/μl, in order to obtain high copy number of plasmids^35^,^36^.

### Expression and purification of the Recombinant Synthetic Antigen

The RSA was cloned in the expression vector pLATE 31, from aLICator LIC Cloning and Expression Kit 3 (#K1261, Thermo Scientific^®^), according to manufacturer’s instructions. This vector was used to clone CaCl_2_ competent *E. coli* BL21 Star (DE3) cells by heat shock. The process enabled the synthetic production of the RSA with a polyhistidine tail end (6xHis), allowing its purification by immobilized metal-ion affinity chromatography (IMAC) with Nickel ions^37^.

The transformed *E. coli* BL21 Star (DE3) cells were grown at 37 °C in Luria-Bertani (LB) broth containing 50 mg/ml of ampicillin, until an absorbance of 0.5 at 600 nm. The RSA expression was then induced by incubation with isopropyl-β-D-thiogalactopyranoside (IPTG) at a final concentration of 1 mM (3 h at 37 °C). An expression control for each colony was created in the absence of IPTG. The pellets were harvested by centrifugation at 5000 *g* for 5 minutes at room temperature after the induction period.

For purification of the RSA, the cell pellets from 15 ml of induced cultures were resuspended in 6 ml of lysis buffer (20 mM NaH_2_PO_4_, 1 mM DTT, 20 mM imidazole, 0.1 mM PMSF, 0.5 M NaCl, 0.2% Triton X-100) for 10 minutes and then subjected to a thermal shock with three cycles of boiling (95–100 °C) for 5 minutes, followed by cooling on ice (0 °C) for 5 minutes. The supernatants were recovered by centrifugation at 15,700 *g* for 5 minutes.

The supernatants were transferred into a mini-column polyprep (Biorad^®^), prepared with a histidine chelating resin with a ratio of 6 mL of sample to 1 mL of resin 50% (Ni Sepharose™ 6 Fast Flow, GE Healthcare^®^), and incubated for 2 h at room temperature. The column was washed three times with 1 ml of ligation buffer (20 mM NaH_2_PO_4_, 0.5 mM NaCl, 20 mM imidazole [pH = 7.4]), and the recombinant protein was then eluted eight times with 0.5 ml of elution buffer (20 mM NaH_2_PO_4_, 0.5 mM NaCl, 500 mM imidazole [pH = 7.4]). The eluted proteins were desalted with a desalting membrane (D-0655, Sigma^®^), as described elsewhere^28^. The protein concentration of the RSA was determined (Nanodrop 1000, Thermo Scientific^®^) and a final concentration of 10 μg/ml was obtained by diluting the RSA (1:40) with sodium bicarbonate (NaHCO_3_, 50 mM [pH 8.4]).

### SDS-PAGE and ELISA analysis

The eluted RSA was analysed by sodium dodecyl sulphate-polyacrylamide gel electrophoresis (SDS-PAGE) on 15% acrylamide gels (mini-PROTEAN electrophoresis cell, Biorad^®^; EC4000P, Apparatus^®^) and by indirect ELISA, using anti-polyhistidine antibodies, in order to confirm the protein expression and purification of the antigen.

The purified RSA was applied as a tool in an indirect ELISA technique. The ELISA was optimized for detection of Ig, IgG and IgM antibodies anti-*P. jirovecii*. In an ELISA plate (Greiner^®^), 50 μl of the RSA was added to the flat transparent wells. The synthetic antigen was coated to the plate for 18 hours at 4 °C. The plate was washed with PBS and 70 μL of 1% PVA were added to the wells and incubated for 1 hour at room temperature (20–25 °C). After blocking, PVA was removed from the plate without washing. Then, the plate was incubated with 50 μl of serum samples (1:80 dilution in PBS with 0.05% Tween 20 and 0.5% BSA) at 37 °C for 1 hour. The plate was washed (three times) with washing buffer PBS–Tween 20 ([PBS-T] - 8 g NaCl; 0.2 g KCl; 1.44 g Na_2_HPO_4_; 0.24 g KH_2_PO_4_ {pH 7.4}; 0.05% Tween 20) and with distilled water (one time). At that point, the specific anti-human Ig (anti-human immunoglobulin [A3813 1:10000 diluted, Sigma^®^], or anti-human immunoglobulin M [2020-04 1:3000 diluted, Sigma^®^], or anti-human immunoglobulin G [2040-04 1:3000 diluted, Sigma^®^]) conjugated to alkaline phosphatase, was added to the wells and incubated for 1 hour at 37 °C. After washing, colour was developed by the addition of 50 μl of the substrate solution containing 4-nitrophenylphosphate sodium salt (10 mg/mL, AppliChem^®^). The plate was incubated for 1 hour at room temperature and the optical densities were measured at 405 nm with an automatic microplate reader (Infinite 200 Pro, Tecan^®^). ELISA results were determined for each serum sample in duplicate.

### Statistical analysis of data

The analysis of the data allowed to evaluate the reliability of the ELISA developed, using statistical measures such as sensitivity, specificity, negative and positive predictive values and Receiver Operator Curves (ROC). The chi-square test (χ^2^) and Fisher’s exact test were used, to study the association between two qualitative variables. The Mann-Whitney test was applied to determine the difference between the distributions of values of the different antibodies used, when we compared two groups of patients. This test was replaced by Kruskal-Wallis test, when comparing more than two groups of patients. Statistical tests were applied with a confidence level of 95%. The Statistical Package for Social Sciences (SPSS) version 20.0 (SPSS Inc., Chicago, IL, USA) was used to perform the statistical analysis.

## Results

### Sampling characterization

This study analysed serum specimens from a universe of 105 persons who were previously categorized into five groups. 50 serum specimens from patients with PcP (47.62%), 11 serum specimens from patients colonized with *P. jirovecii* (10.48%), 19 serum specimens from patients without *P. jirovecii* or other fungal infections (18.09%), eight serum specimens from patients without *P. jirovecii* infection but with other fungal diseases (7.62%) and 17 serum samples from blood donors (16.19%), were analysed (Table 1).

### Recombinant synthetic antigen production and purification

**Figure 1:**
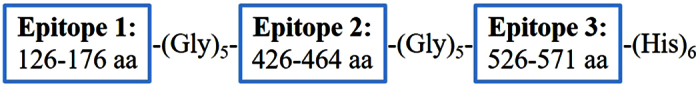
Msg RSA. Representation of the multi-epitope RSA of *P. jirovecii’s* Msg designed in this study. *MSG*_1696–1851_ (126–176 aa) is located at the terminal fraction of the MsgB portion, while *MSG*_2596–2712_ (426–464 aa) and *MSG*_2896–3033_ (526–571) are integrated at the C-termini of the MsgC portion, according to the sequence with GenBank accession no. JN792933.1.

A sequence of 582 amino acids corresponding to the terminal fraction of the Msg of *P. jirovecii* (assembling the terminal portion of the MsgB fraction and the entire MsgC fragment) was assessed.Through the *in silico* analysis of hydrophilicity, accessibility, flexibility, secondary structure, and polarity for this specific sequence, three potential epitopes with high predicted antigenicity and reactivity were selected (Fig. 1). The three potential immunogenic regions selected, Msg_1696–1851_ (126–176 aa) which is located at the terminal fraction of the MsgB portion, Msg_2596–2712_ (426–464 aa) and Msg_2896–3033_ (526–571 aa), both integrated at the C-termini of the MsgC portion, were identified according to the sequence with *GenBank* accession no. JN792933.1 (see supplemental Figures S1–3). This three regions presented high hydrophilic profiles and were located at relatively conserved regions of the Msg sequence.

The *P. jirovecii* multi-epitope RSA designed is composed by 152 amino acids (136 aa from the three selected regions of the Msg original sequence, 10 glycines from the two ligation bridges residues and six histidines from the polyhistidine tail end), have a molecular weight of 16.7 kDa and an isoelectric point of 8.57. The online tool ExPASy – ProtParam characterizes this RSA as stable, with an estimated half-life in *E. coli* higher than 10 hours and a hydropathicity index of −0.702.

After expression of the RSA by *E. coli* BL21 Star (DE3) cells, an electrophoresis of proteins by SDS-PAGE was carried out. The stimulated bacteria expressed a fragment corresponding to the RSA size (16.7 kDa) and this fragment was absent in the control cells.

The RSA was then purified by IMAC and the products were analysed by SDS-PAGE. The products obtained in the elution buffer fractions, showed intense bands corresponding to the molecular weight of the *P. jirovecii* RSA.

### Detection/quantification of anti-*P. jirovecii* antibodies in human serum

**Figure 2:**
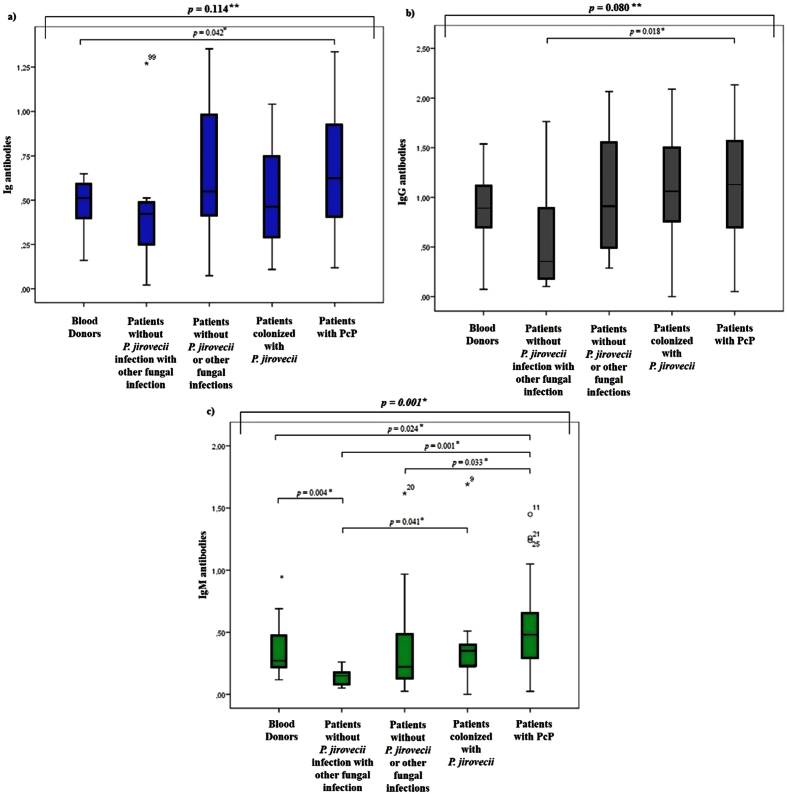
Statistical analysis of the antibodies distribution. Box-and-whisker plots showing the levels distribution of Ig [(**a**)], IgG [(**b**)] and IgM [(**c**)] antibodies anti-*P. jirovecii* in serum of different patient groups studied, with representation of the statistic value (*p*) with no statistical significance (**) and with statistical significance (*).

The serum samples were tested by ELISA. Therefore, each sample was subjected to three separate ELISA tests for detecting human anti-*P. jirovecii* antibodies: Ig, IgG and IgM. Figure 2 depicts the statistical analysis testing the differences in the distribution of the medians obtained in ELISA assays between the groups of patients studied.

The medians distribution of the different antibodies studied by ELISA were also analysed according to the parasite load and the immunosuppression status of the subjects. It was observed an increase in IgM antibodies with increased parasitic load (*p* = 0.004). It was also observed that the distribution of the IgM class varies between HIV- and non-HIV-infected patients (*p* = 0.003), being more pronounced in the HIV-infected ones.

**Figure 3:**
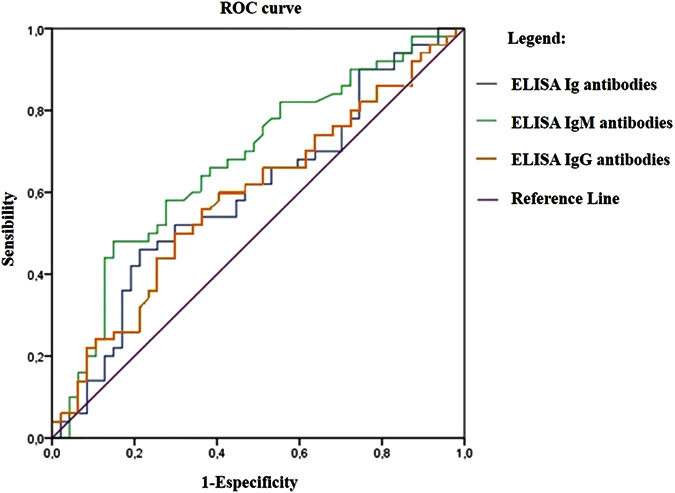
ROC curves of ELISA assays. Representation of ROC curves for the three different ELISA assays (Ig, IgM and IgG antibodies) performed in this study. Areas under the curve were determined with a 95% confidence interval and were 61.2% for total antibodies (*p* = 0.047), 70.7% for IgM antibodies (*p* < 0.001) and 61.4% for IgG antibodies (*p* = 0.044). All the results were statistically significant (*p* < 0.05), rejecting the hypothesis that the distribution of these antibodies is the same for patients with and without PcP.

Figure 3 shows a comparative study of the performance of the three ELISA assays, by representing the ROC curve for each ELISA test. The ROC curves analysis, demonstrates that the ELISA IgM anti-*P. jirovecii* test scored the most promising results. To determine the optimal cut-off limit of this test for the diagnosis of PcP, four different cut-off values were screened and evaluated, as described in Table 2.

The ELISA IgM anti-*P. jirovecii* assay showed an optimal cut-off of 0.350 Abs at 405 nm, with 68% sensitivity and 61.8% specificity (*p* = 0.002). However, these values alone do not exhibit reliability for the serological diagnosis of PcP. Therefore, the diagnostic usefulness of the IgM anti-P. *jirovecii* indirect ELISA was studied in association with the clinical diagnostic criteria for PcP: a PcP case was defined as a patient that met the clinical diagnostic criteria for PcP, plus had anti-*P. jirovecii* IgM antibodies levels equal to or higher than 0.35 absorbance units (AU). A PcP-negative case was defined as a patient that did not met the clinical diagnostic criteria for PcP and demonstrated anti-P. *jirovecii* IgM antibodies levels lower than 0.35 AU. A case was labelled undetermined when the clinical parameters and the anti-*P. jirovecii* IgM antibodies test yielded contradictory results. The sensibility, specificity, positive and negative predictive values were assessed based on cases with agreement between ELISA test result and clinical diagnosis (Table 3).

## Discussion

Currently, the definitive diagnosis of PcP involves the direct identification of *P. jirovecii* in respiratory specimens, particularly in BAL, the standard biological specimen, and therefore the most used. BAL is obtained by performing an expensive and invasive technique (bronchoscopy) that often is difficult to perform in patients with respiratory failure or in children^6^. In this perspective, it is urgent to develop a method minimally invasive, cost-effective, accurate and based on serological biomarkers, for diagnosis of PcP infection.

Serum LDH enzyme, the β-glucan structural component, antigen KL-6 and S-adenosylmethionine (SAM), have been associated with PcP^7^,^8^,^9^,^10^,^11^,^12^,^13^,^14^,^15^. However, despite their utility, all these serological markers are not narrowly specific for *P. jirovecii* infection. The LDH may be increased in any other pathology with cell damage, while β-glucan, as a structural component of fungi, may be increased in patients with other fungal infections. The KL-6 antigen, being a constituent of the alveolar type II pneumocytes, may also change its levels in any other pathology that causes injury to the lung parenchyma. Moreover, the applicability of serum levels of SAM are raising contention among various authors, not being well defined the relationship between the variation of serum levels of SAM and the infection caused by *P. jirovecii*. On top of this, even using the β-glucan, which is the serological marker that has shown more promising results, there is still no consensus cut-off for the diagnosis of PcP^14^.

In the state of art of serological diagnosis of PcP, different authors have studied the immune response of patients with and without PcP with different fractions of the recombinant protein Msg of *P. jirovecii*^16^,^17^,^18^,^19^,^20^,^21^,^22^,^23^,^24^,^25^,^26^. Although there are an estimated 100 related but unique copies of the *MSG* gene in the *Pneumocystis* genome, a greater variability is notice in the amino-terminus of the Msg protein and the most conserved sequences are present at the carboxyl-terminus. Therefore, the carboxyl-terminus, especially the MsgC portion, appears to be the most useful portion for application in the immunodiagnosis and epidemiological studies of PcP^9^,^10^,^11^,^12^,^13^,^14^,^15^,^16^,^17^,^18^,^16^,^23^,^24^,^34^. However, an approach where targeting antigenic regions of the terminal portion of the Msg protein to improve discrimination between patients with and without PcP, was never tried before. Therefore, we designed a Msg RSA composed by three potential epitopes with high predicted antigenicity and reactivity (see supplemental Figures S4–S9 and Table S1). To ensure higher coverage of the potential epitopes existing in the terminal portion of Msg, the fraction between the amino acids 441–1022 was considered for the design and construction of the final RSA. This specific fraction of the *MSG* gene (between nucleotides 1321–3192) englobes the terminal sequence of the MsgB portion and the entire MsgC portion.

The selection of the three specific epitopes was based on the compartmental analysis of each theoretical reactive region, and the most hydrophilic, antigenic, flexible and reactive ones were considered potential antigenic epitopes. On the other hand, the selected epitopes were located at relative conserved regions as can be seen in the supplementary Figure S3. Besides this, epitopes nearby the C-termini of the MsgC protein were preferably chosen, because C-termini of proteins are often exposed and have a high degree of flexibility, making them usually a good choice for generating anti-peptide antibodies directed against the intact protein^38^. In summary, the selected potential reactive epitopes of the RSA present high predicted antigenicity and reactivity, are located in relatively conserved regions and represent the terminal fraction of the MsgB and the C-termini of the MsgC.

Although other potential antigenic epitopes were widely distributed along the entire amino acid sequence of the MsgC fragment, none were chosen because we were looking for a small and simple RSA, to make an initial assessment of the utility of this approach for the serological diagnosis of PPc.

On the other hand, since few previous studies analysed IgG and IgM fractions produced against recombinant fractions of Msg, in this study, ELISA assays were developed to attempt to understand if this RSA can be recognized by human antibodies (Ig, IgG and IgM), allowing the discrimination of persons with and without the disease. In the analysis of Fig. 2a, it can be observed an increase in Ig antibody levels of patients with PcP. However, this increase was not statistically significant (*p* = 0.114) compared to the Ig antibodies medians of the other groups. Yet, there was a significant difference between the median values of blood donors’ total antibodies and PcP patients, so the application of this test may be useful in distinguishing healthy persons from PcP patients (*p* = 0.042). In Fig. 2b, there was an increase in production of IgG in patients with PcP, however the difference to the value recorded in other groups was not statistically significant (*p* = 0.080). Though, there was statistical significance between the IgG median values of patients with other fungal pathologies and patients with PcP (*p* = 0.018), demonstrating the potential utility of this serological marker as a discriminatory tool between *Pneumocystis* and other fungal infections. In Fig. 2c, there was a statistically significant difference (*p* = 0.001) between the IgM antibodies median values among the five groups analysed, which indicates that the distribution of the IgM class is not random between different clinical groups. More important was the existence of statistical significance between the IgM median of PcP patients compared to healthy persons and the other patients groups, showing that this test has application in the discrimination of these different clinical conditions.

The absence of statistical significance between the medians of the Ig antibodies anti-*P. jirovecii* of the different groups, could be due to the lack of significance between IgG medians values, which compromised the results obtained with Ig antibodies. However, these results go against recent studies in which IgG levels to various recombinant fractions of MsgC were higher in HIV-infected patients with a history of PcP, compared to patients with no clinical history of PcP^16^,^18^,^19^,^21^,^22^,^23^,^24^. This circumstance may be due to the fact that the region analysed in this study was not identical to the regions examined by other authors, since we synthetized a RSA of the terminal portion of Msg. Moreover, the lack of sensitivity for detection of IgG antibodies by our ELISA technique may also explain these differences. Yet, the present study shows that there is indeed a difference in the levels of IgM antibodies between patients with PcP and the other groups analysed.

Figure 3 shows the ROC curves, the graphic representation of the true positive rate against the false positive rate for the different cut-offs of a test. In this study, despite all ELISA tests have achieved an area under the curve (AUC) with statistical significance (p < 0.05), the IgM anti-*P. jirovecii* assay is the one with the most promising result (p < 0.001), presenting an AUC of 70.7%. Then, the ELISA test with detection of IgM antibodies anti-*P. jirovecii* seems to show some applicability to the serological diagnosis of PcP and so we needed to test its reliability and sturdiness. An ideal test is the one that is able to identify all cases of disease being highly sensitive. At the same time, a perfect test should still be able to correctly identify all the people who do not have the disease, being specific to the pathology. Therefore, a cut-off value for this ELISA test, that balances the sensitivity and specificity needed, was chosen (Table 2). The results obtained for the different cut-off values confirmed that the best balance between sensibility and specificity was recorded at the cut-off value of 0.350 AU. With this cut-off value, the test will be negative in 61.8% of patients without PcP and it will be positive in 68% of patients with PcP. However, this cut-off value, as well as the other three studied, show no statistical measures of excellence that allow us to ensure a serological diagnosis of PcP with complete confidence. Yet, the data presented in Table 3, where the results of the ELISA test for IgM antibodies anti-*P. jirovecii* were associated with the clinical diagnosis of PcP of each patient, showed promising results for the serological diagnosis of PcP.

If we look at this association as a screening test, we can classify the patients to the condition of having or not PcP, with a sensitivity of 100% and a specificity of 80.8%. The positive and negative predictive values stood at 87.5% and 100%, respectively, and the statistic test leaves no doubt about the dependency of the results of the ELISA assay and the clinical diagnosis. Thus, the implementation of this test as a screening test could contribute to an improvement in health care, reducing the practice of empirical therapy, especially in low-middle income countries, in patients with respiratory failure and in children, in whom the execution of invasive techniques such as bronchoscopy are not easy to accomplish. Moreover, the association of the ELISA IgM anti-*P. jirovecii* assay with the clinical diagnosis of patients with suspected PcP, show great applicability in epidemiological studies. It will be possible to evaluate the population’s immunity against the etiological agent of PcP, using a specific biosensor of *P. jirovecii* and a biological specimen (blood) obtained by a minimally invasive technique, sensitive and specific in diagnosing the disease.

With this innovative approach, we succeeded in producing a recombinant synthetic antigen, specific for *P. jirovecii*, capable of functioning as a PcP biosensor when applied in an ELISA assay for detection of IgM antibodies anti-*P. jirovecii* (patent-pending no. PT109078) and when associated with the clinical diagnosis of each patient. This new method may be used as a screening test for PcP, decreasing the need for biological specimens obtained by invasive techniques, which is a major benefit to the patient’s care and an improvement in the clinical management of the disease.

## Additional Information

**How to cite this article**: Tomás, A. L. *et al*. Serological diagnosis of pneumocystosis: production of a synthetic recombinant antigen for immunodetection of *Pneumocystis jirovecii. Sci. Rep.* **6**, 36287; doi: 10.1038/srep36287 (2016).

**Publisher’s note:** Springer Nature remains neutral with regard to jurisdictional claims in published maps and institutional affiliations.

## Supplementary Material

Supplementary Information

## Figures and Tables

**Table 1:** Clinical, immunological and laboratory information of the 88 patients previously studied, with suspicion of PcP.

Parameters	Number of patients (%)
Immunodeficiency state	
HIV-infected	75 (85.23)
Organ/marrow transplanted	6 (6.82)
Cancer	7 (7.95)
PcP laboratory diagnosis	
IF/Mab+	28 (35)
PCR+	61 (75)
IF/PCR+	28 (35)
Fungal burden	
Negative for *P. jirovecii*	27 (30.7)
Low/Moderate	49 (55.7)
High	12 (13.6)
PcP final diagnosis	
Patient with PcP	50 (56.82)
Patient colonized with *P. jirovecii*	11 (12.5)
Patient without *P. jirovecii* or other fungal infection	19 (21.59)
Patient without *P. jirovecii* infection with other fungal infection	8 (9.09)

**Table 2:** Statistical measures calculated for different cut-offs studied.

Statistical measures	Cut-off values			
	0.250 (Abs)	0.286 (Abs)	0.300 (Abs)	0.350 (Abs)
Sensibility (%)	82.0%	76.0%	72.0%	68.0%
Specificity (%)	52.7%	56.4%	58.2%	61.8%
Positive predictive value (%)	61.2%	61.3%	61.0%	61.8%
Negative predictive value (%)	76.3%	72.1%	69.6%	68%
Chi-square *p* value	0.001	0.001	0.002	0.002

**Table 3:** Statistical measures for the association of ELISA IgM anti-*P. jirovecii* test results with the patient clinical diagnoses of PcP.

Statistical measures	Cut-off
	0.350 (Abs)
Sensibility (%)	100
Specificity (%)	80.8
Positive predictive value (%)	87.5
Negative predictive value (%)	100
Chi-square *p* value	<0.001
Cases with agreement between ELISA test result and clinical diagnosis (%)	58.1
