# Supplementary material for: Serological diagnosis of pneumocystosis: production of a synthetic recombinant antigen for immunodetection of Pneumocystis jirovecii
Source: Sci Rep. 2016 Nov 8;6:36287. doi: 10.1038/srep36287 (PMC5099754; doi:10.1038/srep36287)
Supplement: Supplementary Information [file srep36287-s1.pdf]

Supplementary information

Serological diagnosis of pneumocystosis: production of a synthetic recombinant antigen for immunodetection of *Pneumocystis jirovecii*.

Tomás AL[1], Cardoso F[1], Esteves F[2] and Matos O[1*];

[1] Unidade de Parasitologia Médica, Grupo de Protozoários Oportunistas/VIH e Outros Protozoários, Instituto de Higiene e Medicina Tropical, Global Health and Tropical Medicine, Universidade Nova de Lisboa, Lisboa, Portugal.

[2] Centro de Toxicogenómica e Saúde Humana (ToxOmics), Departamento de Genética, NOVA Medical School/Faculdade de Ciências Médicas, Universidade Nova de Lisboa, Lisboa, Portugal.

[*] Electronic address: omatos@ihmt.unl.pt.

Figures

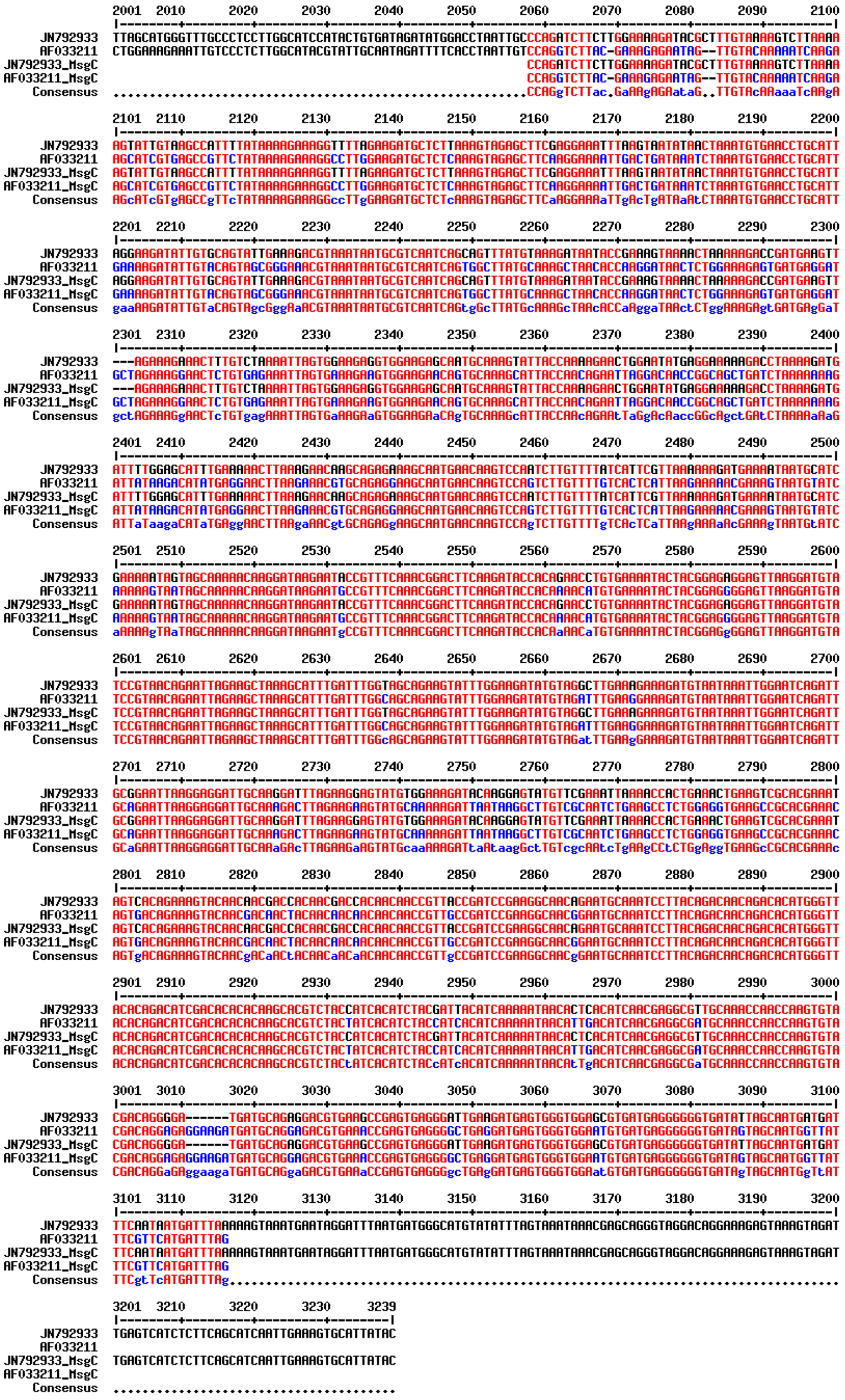

Supplementary figure S1. Alignment of the nucleotide sequence of the MsgC portion from GenBank AF033211 and JN792933.1. This alignment allows to locate the nucleotides corresponding to the MsgC portion in the sequence selected for the design of the RSA (JN792933.1) (Multalin version 5.4.1).

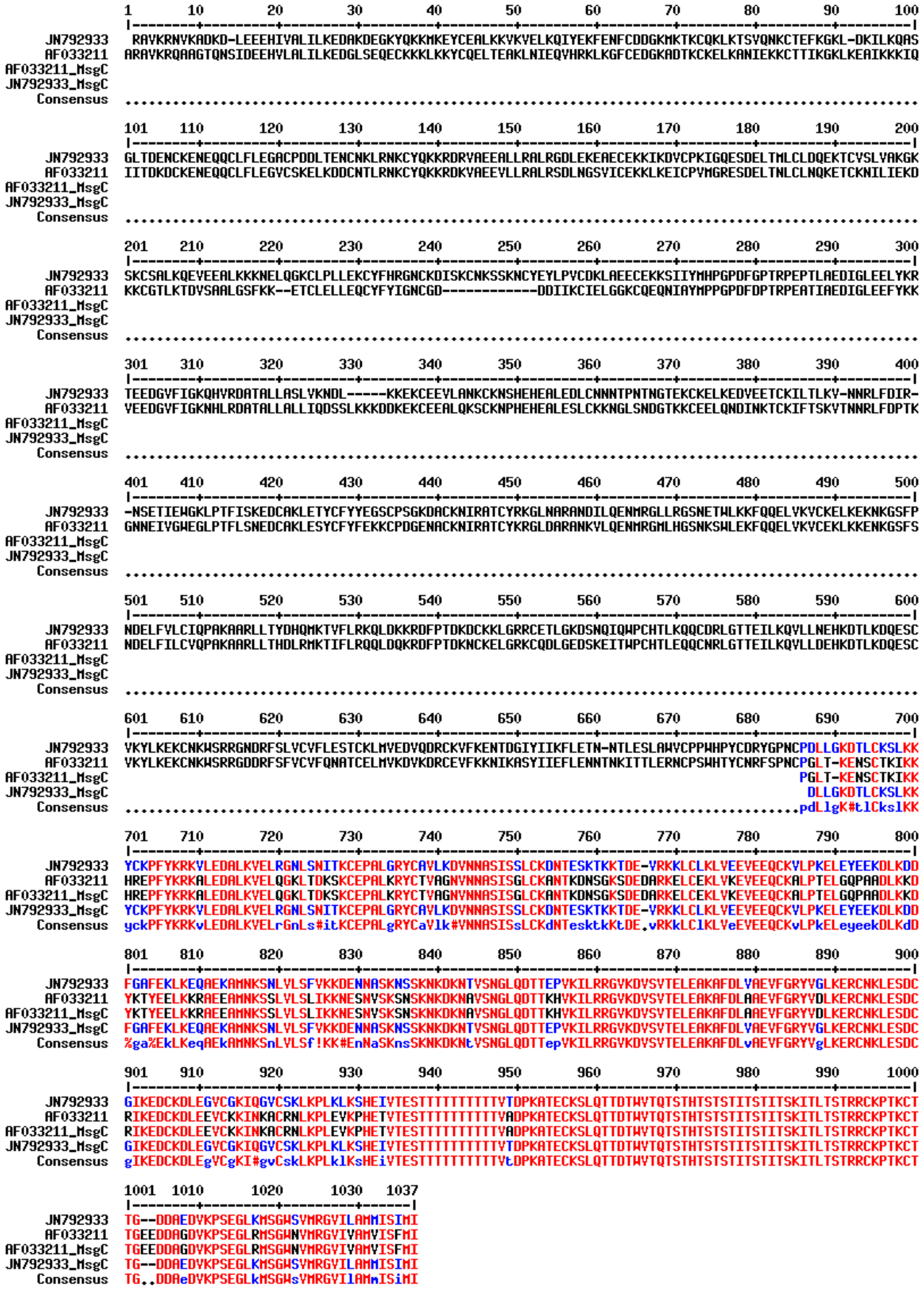

Supplementary figure S2. Alignment of the amino acid sequences of the MsgC from GenBank AF033211 and JN792933.1. This alignment allows the discrimination of the most conserved regions (Multalin version 5.4.1).

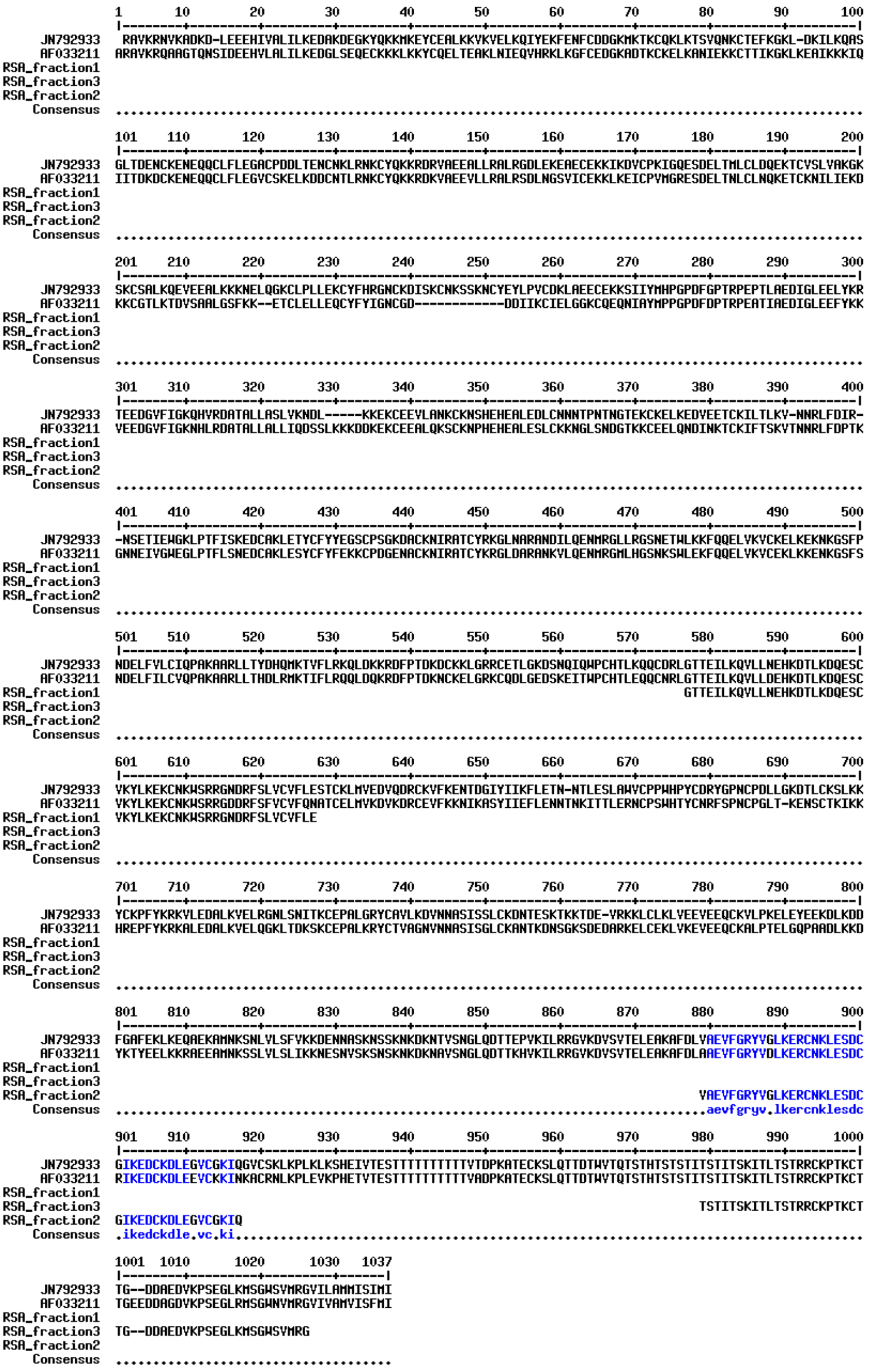

Supplementary figure S3. Alignment of the amino acid sequences of the Msg from GenBank AF033211 and JN792933.1 and the three amino acid sequences of the regions selected to produce the final Msg RSA. This alignment shows that the Msg selected regions (region1: 126-176 aa; region 2: 426-464 aa; region 3: 526-571 aa) are located at relative conserved regions (Multalin version 5.4.1).

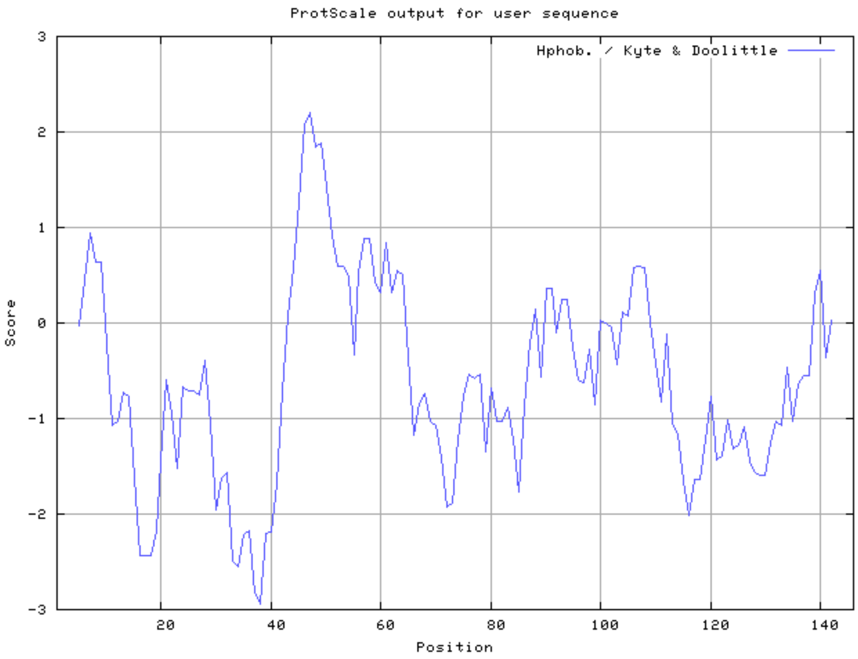

Supplementary figure S4. Representation of hydrophilic behaviour of the RSA designed according to Kyle & Doolittle hydropathic scale. The majority of the amino acid sequence of the RSA have a hydrophobic profile, as was intended (ExPASy – ProtScale).

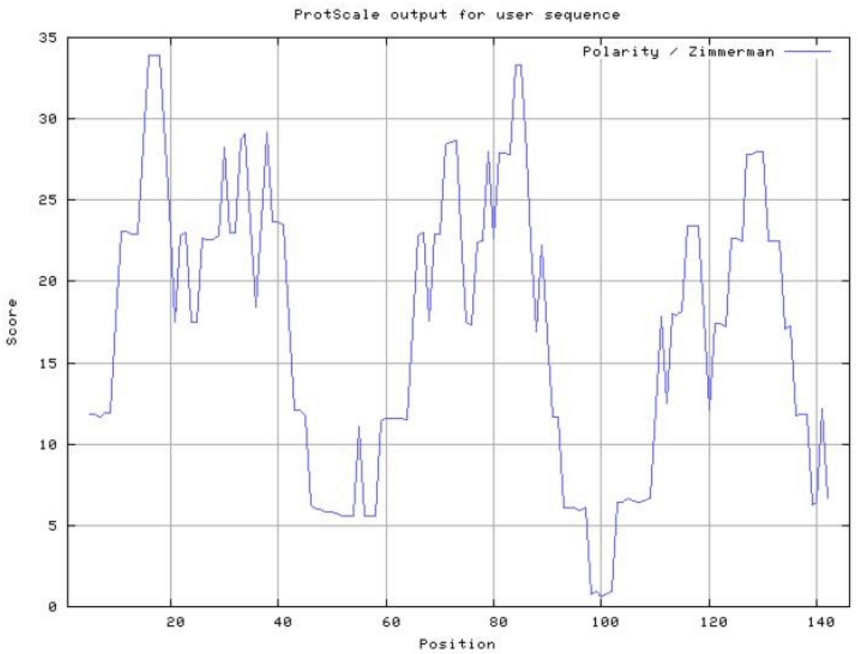

Supplementary figure S5. Representation of polarity of the RSA designed according to Zimmerman scale. The majority of the amino acid sequence of the RSA have a polar profile, as was intended (ExPASy – ProtScale).

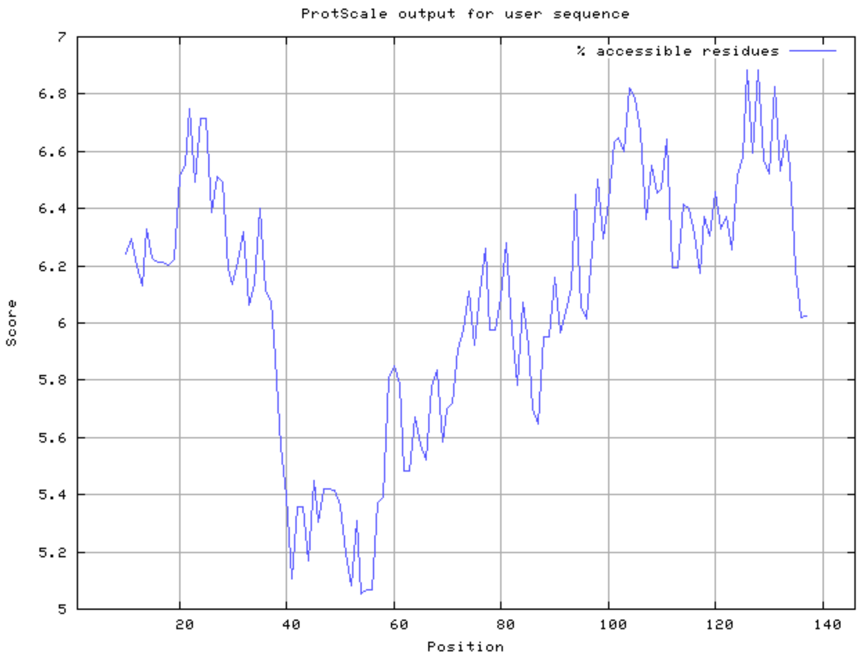

Supplementary figure S6. Representation of accessibility of the residues of the RSA designed. All the amino acid sequence of the RSA have an accessible profile, as was intended (ExPASy – ProtScale).

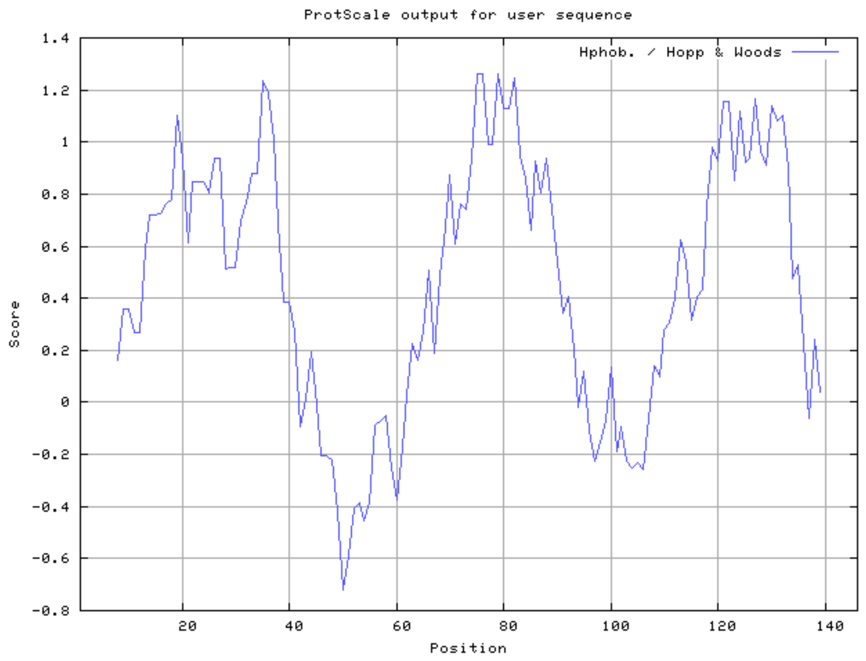

Supplementary figure S7. Representation of antigenicity profile of the RSA designed according to Hopp & Woods scale. The residues of the three epitopes selected have an antigenic profile while the glycine bridges have a less antigenic behaviour, as was intended (ExPASy – ProtScale).

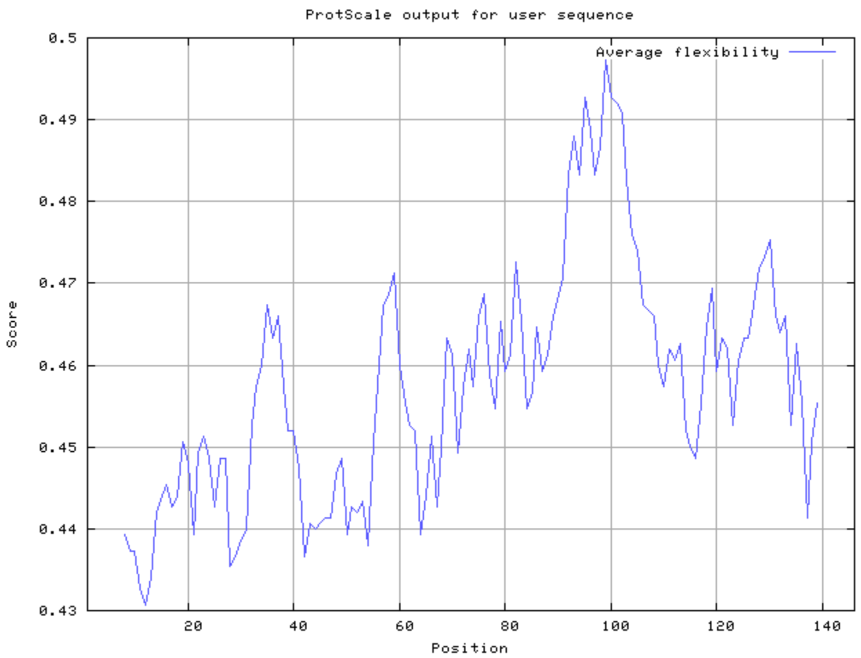

Supplementary figure S8. Representation of the average flexibility profile of the residues of the RSA designed. The residues have a positive average flexibility, as was intended (ExPASy – ProtScale).

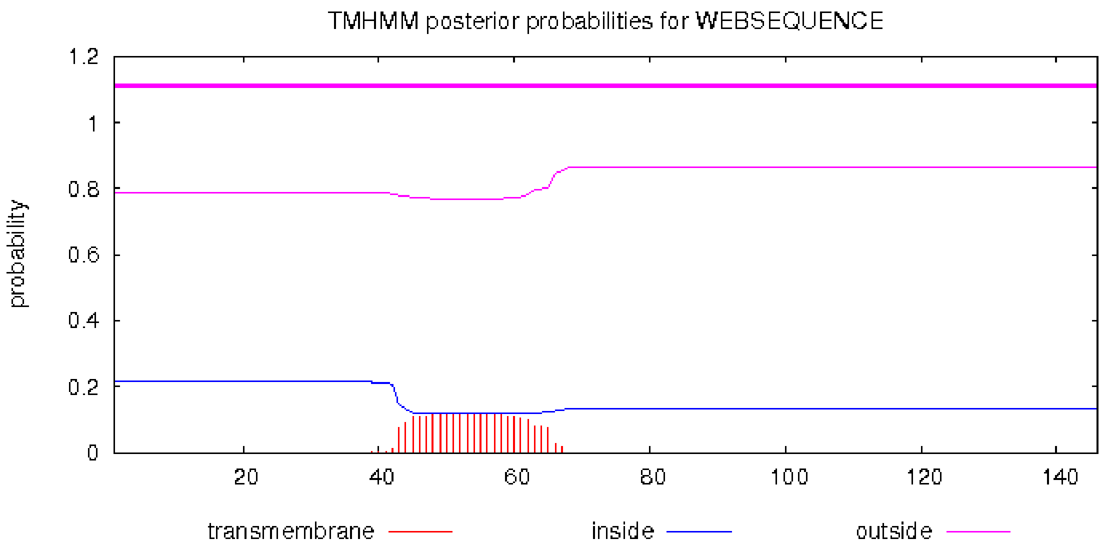

Supplementary figure S9. Representation of the putative position on the cytoplasmic membrane of the amino acids present in the RSA designed, through *CBS – TMHMM* – version 2.0 software. This figure pointed to a probability close to one for an external exposure over the whole region under consideration.

Tables

Supplementary table S1. GOR IV secondary structure prediction of the RSA (NPS@: Network Protein Sequence Analysis).

Structure	Percentage
**Alpha helix**	13.70%
**Extended strand**	27,40%
**Random coil**	58,90%
